# Influence of Artificial Light at Night on Thyroid Gland Histology in *Triturus* Newts (Urodela, Salamandridae)

**DOI:** 10.3390/ani16030483

**Published:** 2026-02-04

**Authors:** Maja Ajduković, Marija Drobnjaković, Branka Šošić-Jurjević, Tijana Vučić, Tamara Petrović, Marko Prokić

**Affiliations:** 1 Department of Evolutionary Biology, Institute for Biological Research “Siniša Stanković”, National Institute of the Republic of Serbia, University of Belgrade, Bulevar Despota Stefana 142, 11000 Belgrade, Serbia; 2 Department of Cytology, Institute for Biological Research “Siniša Stanković”, National Institute of the Republic of Serbia, University of Belgrade, 11000 Belgrade, Serbia; 3 Institute of Zoology, Faculty of Biology, University of Belgrade, Studentski trg 16, 11000 Belgrade, Serbia; 4 Department of Physiology, Institute for Biological Research “Siniša Stanković”, National Institute of Republic of Serbia, University of Belgrade, 11000 Belgrade, Serbia

**Keywords:** ALAN, Caudata, endocrine disruptor, LED, sky glowing, thyroglobulin

## Abstract

Artificial light provides many benefits to humankind, allowing fundamental activities to continue during night; however, it also poses multiple risks to humans and wildlife and is recognized as a significant driver of global environmental change. Changes in natural light/darkness cycles caused by artificial light at night (ALAN) can affect amphibians, the most threatened vertebrate group globally. The aim of this study was to determine the effects of long-term exposure to constant nighttime light on the morphology of the thyroid glands of *Triturus ivanbureschi* metamorphosed juveniles using histological analysis. A cool LED light with a color temperature of 6000 K was selected, as this spectrum is commonly used in outdoor lighting. Larvae were raised at a natural day–night light regime. After metamorphosis, juveniles were randomly divided into a control group maintained under natural dark nighttime conditions (<0.1 lux) and a treatment group exposed to LED light (30 lux) at night for 60 days. Standard histological techniques (H&E) and immunohistochemical (IHC) staining were used to examine the thyroid glands. There were no significant differences in the absolute volume densities between the light-treatment and control groups; however, subtle morphological variations were observed. Immunohistochemical analysis of Tg immunostaining revealed a significant difference between the light-treatment and control groups, indicating that the thyroid gland of newts exposed to light has a stronger signal, suggesting the accumulation of thyroglobulin at the apical surface of the follicular cells. As LED lighting continues to expand globally, understanding how different light spectra, intensities, and exposure durations influence thyroid function, particularly during early life stages, remains an important direction for future research.

## Simple Summary

Artificial light, while beneficial for human activities at night, poses potential health risks to humans and wildlife, particularly affecting amphibians, which are experiencing a global population decline. This study investigates the impact of artificial light at night on the thyroid gland morphology of *Triturus ivanbureschi* metamorphosed juveniles. Using LED lighting, juveniles were assigned to a control group with dark night conditions and a treatment group maintained under a light intensity of 30 lux for 60 days. Histological analysis revealed no significant differences in the absolute volume densities of the thyroid gland between groups, as well as in the volumes of the follicular epithelium, colloid, and interstitium, although some morphological variations were noted. Control thyroid glands showed uniformity, smaller follicles, and signs of active secretion, while light-exposed glands displayed macrofollicles with a less active state. Immunohistochemical analysis indicated stronger thyroglobulin signals in the light-treatment group, suggesting accumulation at the follicular cell surface and a potential hypofunctional thyroid state. These findings imply that artificial light at night may disrupt endocrine function and affect amphibian thyroid gland morphology and secretion. Further molecular research is needed to understand the physiological implications of these structural changes.

## 1. Introduction

Artificial light at night (ALAN) is a major driver of global nighttime sky brightening. During the last century, the introduction of manmade light started to illuminate the nocturnal environment, eroding natural light cycles. This process intensified during the past decade following a drastic increase in urbanization, socio-economic development and installation of light-emitting diodes (LEDs) technology [1]. With increasing rates of 9.6% per year on a global level, artificial light has become one of the fastest-spreading environmental changes in the Anthropocene [2]. Today there are large areas of the Earth, including almost half of the USA and a majority of Europe (about 88%), experiencing brightness levels higher than natural thresholds [3,4]. It was shown that in humans, exposure to extensive ALAN is linked to a range of health effects, including sleep disturbances, circadian rhythm disruption, melatonin suppression, and an increased risk of metabolic disorders, depression, and certain types of cancer [5,6,7]. A notable study demonstrates a correlation between ALAN and thyroid cancer in humans [8]. Individuals residing in areas with the highest ALAN exposure have a higher risk of developing thyroid cancer compared to those in the lowest quintile, potentially through a mechanism involving disruption of circadian rhythms and melatonin production. Beside humans, ALAN can have an impact on all vertebrate groups, disturbing ecological processes such as species abundance and distribution and modifying ecosystem functioning [9,10].

Nocturnal species, which represent more than 28% of vertebrate species, are expected to be sensitive to light pollution [11]. Among them are amphibians that are mainly active at night [12]. This nocturnality helps them to avoid predators such as birds and bats and increase their chances of survival. Also, nighttime offers higher humidity and cooler temperatures, which are crucial for preventing desiccation and normal skin respiration during the terrestrial phase [12]. Amphibians often live or breed in wetlands located within or near human settlements, or in areas restored or created for recreational purposes, and as a result they are exposed to artificial light. Because of their low dispersal ability and strong dependence on surrounding habitats, amphibians are less able to avoid changes in nighttime lighting. All these traits put them at risk from exposure to different intensities, sources and timing of ALAN [13]. Previous studies suggest that ALAN can disrupt various physiological processes in amphibians [14,15,16,17].

The thyroid gland in amphibians is a paired structure located in the lower jaw lateral to the geniohyoideus muscles. As in other vertebrates, the thyroid is a highly conserved organ composed of numerous spherical functional units known as follicles. Each follicle consists of a simple epithelial layer surrounding a central lumen filled with a gel-like substance termed colloid [18]. A key constituent of the colloid is thyroglobulin (Tg), a glycoprotein produced by thyroid epithelial cells, secreted apically, and stored within the follicular lumen. Follicles are embedded in an interfollicular connective tissue matrix (interstitium) that contains an extensive network of blood vessels. Thyroid hormones (THs), L-thyroxine (T4) and L-triiodothyronine (T3), are essential regulators of amphibian larval development and body growth and play a central role in metamorphosis. The synthesis and secretion of THs into the circulation progressively increases during development, reaching peak levels at the metamorphic climax [19,20,21]. In juvenile amphibians, thyroid hormones regulate post-metamorphic growth, metabolism, and tissue maturation, as well as seasonal physiological transitions such as preparation for hibernation [22,23]. Endocrine disruptors can alter the normal function of the thyroid gland. Substances such as bisphenol A, phthalates, thiourea, pesticides, and many others mimic natural hormones, block hormone receptors, and alter hormone synthesis, transport and metabolism [24,25,26,27]. In recent years, ALAN is increasingly recognized as one of the potential endocrine disruptors, particularly because of its interference with melatonin production and circadian rhythms, leading to widespread physiological consequences [28,29]. Although ALAN-induced alterations to thyroid gland histology have been documented in mammals, especially rodents [30,31], the potential impacts on amphibian thyroid morphology remain unknown.

Given the absence of direct evidence on how ALAN affects thyroid structure in amphibians, and considering their increased sensitivity to environmental cues, the present study aimed to determine whether chronic nighttime exposure to cool-white LED light alters thyroid gland morphology and thyroglobulin dynamics in metamorphosed juveniles of crested newts (*Triturus ivanbureschi)*. This study provides the first assessment of ALAN-induced thyroid alterations in a urodele species.

## 2. Materials and Methods

### 2.1. Model Species

The genus *Triturus* (Salamandridae) comprises ten species: three marbled (*T. marmoratus*, *T. pygmaeus*, *T. rudolfi*) and seven crested newts (*T. anatolicus*, *T. carnifex*, *T. cristatus*, *T. dobrogicus*, *T. karelinii*, *T. macedonicus*, *T. ivanbureschi*) [32,33,34,35]. In this study we used metamorphosed juveniles of *Triturus ivanbureschi* reared in a laboratory. Eggs were obtained from nine gravid females collected from a natural population from the village Brebevnica (Serbia) (43.02° N, 22.53° E) and brought to the Institute for Biological Research “Siniša Stanković” in Belgrade. The collection of adults from natural populations was approved by the Ministry of Environmental Protection of the Republic of Serbia (permit no. 353-01-1506/2022-04). All experimental procedures were approved by the Ethical Committee of the Institute for Biological Research “Siniša Stanković” (decision no. 323-07-07481/2023-05) and carried out in accordance with European Directive 2010/63/EU.

### 2.2. Animal Housing

To obtain material for this study, gravid females were placed in a tank (200–400 L) filled with tap water and with plastic strips for egg deposition. After the egg-laying period, females were transferred back to the same pond where they were collected. Egg collection, larval rearing under laboratory conditions, and feeding protocols were conducted according to previously published methods [27,36]. Once individuals reached the juvenile stage, identified by complete gill resorption and closure of the gill slits, they were housed individually in 2 L plastic boxes (225 × 175 × 75 mm) filled with dechlorinated tap water and subjected to the experimental procedures. All boxes were placed on shelves in a dedicated dark room. To prevent interference between different light treatments, partitions were installed between shelves assigned to different light regimes. Light intensities were measured repeatedly throughout the experiment to confirm the intended exposure levels. Newts were fed ad libitum with *Tubifex* sp., with food provided directly in the individual boxes. Water was changed every other day, and the temperature was maintained at 18 °C.

### 2.3. Experimental Settings

We used LED light as a predominant outdoor light source, and we chose an intensity of 30 lux, which corresponds to the light intensity perceived in public gardens and emitted by street lights. Besides light intensity, the color temperature of LED light is also important, especially cooler color temperatures (higher than 3000 K), which have more negative effects on nocturnal wildlife [37]. We decided to use a combination of light intensity of 30 lux and color temperature of 6000 K (which are mostly used outdoors).

Metamorphosed juveniles were distributed evenly into a control group (natural darkness at night, <0.1 lux) or a treatment group (30 lux, 6000 K LED light at night) for 60 days. During the day, all individuals were under ~450 lux, 6000 K LED light to simulate natural daylight. An electronic timer controlled the light/dark cycle, which was adjusted weekly to follow the natural photoperiod, decreasing from 13 L:11 D to 11 L:13 D, corresponding to early September and late October in Serbia.

Each experimental group contained six specimens, which represent the minimal number of animals required for histological analyses. The minimal number of animals was used due to the strict protection of *Triturus ivanbureschi* in the Republic of Serbia. This designation has been established under the Regulation on the Proclamation and Protection of Strictly Protected and Protected Wild Species of Plants, Animals, and Fungi, as published in the Official Gazette of the Republic of Serbia (Nos. 5/2010 to 98/2016).

### 2.4. Tissue Preparation and Staining Methods

Following a two-month experimental period, metamorphosed juveniles were humanely euthanized using ethyl 3-aminobenzoate methanesulfonate (MS 222) (CAS number: 886–86–2; Sigma, St. Louis, MO, USA). After euthanasia, the animals were decapitated and the lower jaw was excised and fixed in 4% neutral buffered formaldehyde for 24 h. Procedures for tissue preparation and staining methods for hematoxylin-eosin (H&E) and periodic-acid-Schiff (PAS) followed previously published protocols [27].

Immunohistochemical (IHC) staining was performed following previously published protocols [27]. In this study, Donkey anti-rabbit IgG-horseradish peroxidase (HRP; abcam; 1:200) was applied as a secondary antiserum for 1 h. Digital images were made using a LEITZ DM RB light microscope (Leica Mikroskopie & Systems GmbH, Wetzlar, Germany), with a LEICA DFC320 CCD camera (Leica Microsystems Ltd., Heerbrugg, Switzerland) and Leica DFC Twain Software 7.1.1. (Leica, Wetzlar, Germany).

### 2.5. Stereological Assessment and Quantitative Image Analysis

Stereological measurements were performed on H&E and PAS-stained thyroid sections using a newCAST stereological software package (VIS—Visiopharm Integrator System, version 3.2.7.0; Visiopharm, Hørsholm, Denmark). Absolute thyroid volumes (mm^3^), as well as the volume of thyroid tissue phases (epithelium, colloid and interstitium; mm^3^) were determined using Cavalieri’s principle [38] and an objective magnification of 20× using a test grid (6 × 6). Thyroid volume (V_pt_) was then estimated according to the following formulae:

Vpt = a(p) · d · Σ_i=1_ⁿ Pᵢ

where a(p) is the area associated with each sampling point (12,385.99 μm^2^), dis the block advance representing the mean distance between two consecutively studied sections (25 μm) [39], n is the number of sections studied for each thyroid, and ΣP_i_ is the sum of points hitting a given target. The same sections were used for estimation of absolute thyroid volumes as well as the volumes of thyroid tissue phases: follicular epithelium, colloid and interstitium. The number of thyroids analyzed was 6 per group.

ImageJ Fiji (Version 1.49j) with the open-source IHC Profiler plugin was employed for semi-quantitative analysis of the DAB IHC signal of Tg, allowing for automated assessment of staining intensity [40]. For each animal (four control and four treatment), ten unbiased images (2592 × 1944 pixels, 40× objective) were analyzed. The IHC Profiler plugin performed color deconvolution of the images using preset optical density vectors for DAB and hematoxylin and profiled the DAB-stained cytoplasmic regions. These profiles were then analyzed in ImageJ using the threshold function to determine mean gray intensity. Optical density (OD) was calculated as OD = log (max intensity/mean intensity).

### 2.6. Statistical Analysis

Data normality was assessed using the Shapiro–Wilk test. As the data were not normally distributed, differences between the light-treatment and control groups in the absolute volume density of the thyroid gland and in the volumes of the follicular epithelium, colloid, and interstitium were evaluated using the Mann–Whitney U test. The same test was applied to compare the optical density of Tg immunopositivity between groups. All analyses were performed in Statistica 10 (StatSoft Inc., 2011, Tulsa, OK, USA).

## 3. Results

### 3.1. Histomorphometric Analysis

**Figure 1:**
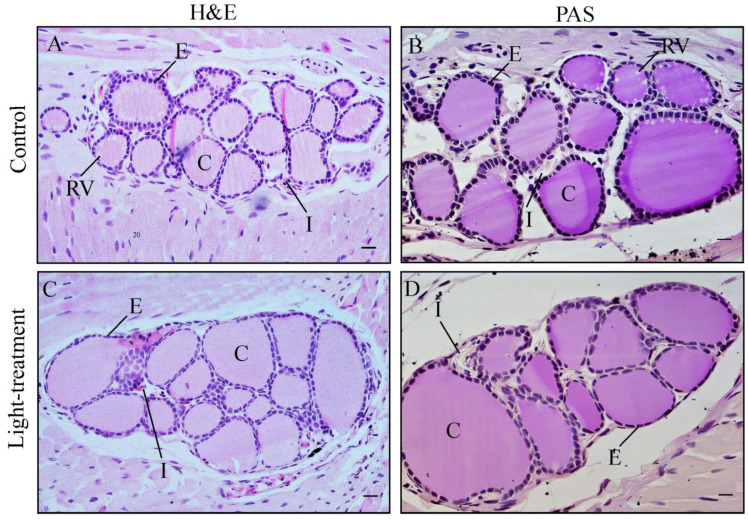
Representative micrographs of the thyroid gland of *Triturus* newts from control and light-treatment groups. Hematoxylin and eosin (H&E) staining, 10× magnification (bar = 200 µm; (**A**,**C**)) and PAS staining, 20× objective magnification (bar = 100 µm; (**B**,**D**)). Abbreviation: C, colloid; E, epithelium; I, interstitium; RV, resorption vacuoles.

In newts, the thyroid gland is a paired, oval structure located in the lower jaw, anterior to the arterial arch and lateral to the geniohyoideus muscles. Each thyroid lobe is composed mainly of thyroid follicles, which consist of follicular epithelium surrounding colloid. Within the colloid, resorption vacuoles are evident, serving as indicators of the activity of the follicular epithelium in the synthesis and secretion of thyroid hormones (Figure 1A,B). Follicles are embedded in interfollicular connective tissue, or interstitium, which is primarily observed as nucleated red blood cells (Figure 1A,D). Such thyroid histology reflects a normal functional state in the control group. Exposure to ALAN induces changes in thyroid tissue, such as the presence of large macrofollicles filled with colloid and surrounded by flattened follicular epithelium (Figure 1C,D). The absence of resorption vacuoles was evident in light-treatment group (Figure 1C,D). Interstitial tissue was also present between the follicles in the light-treatment group (Figure 1D).

**Figure 2:**
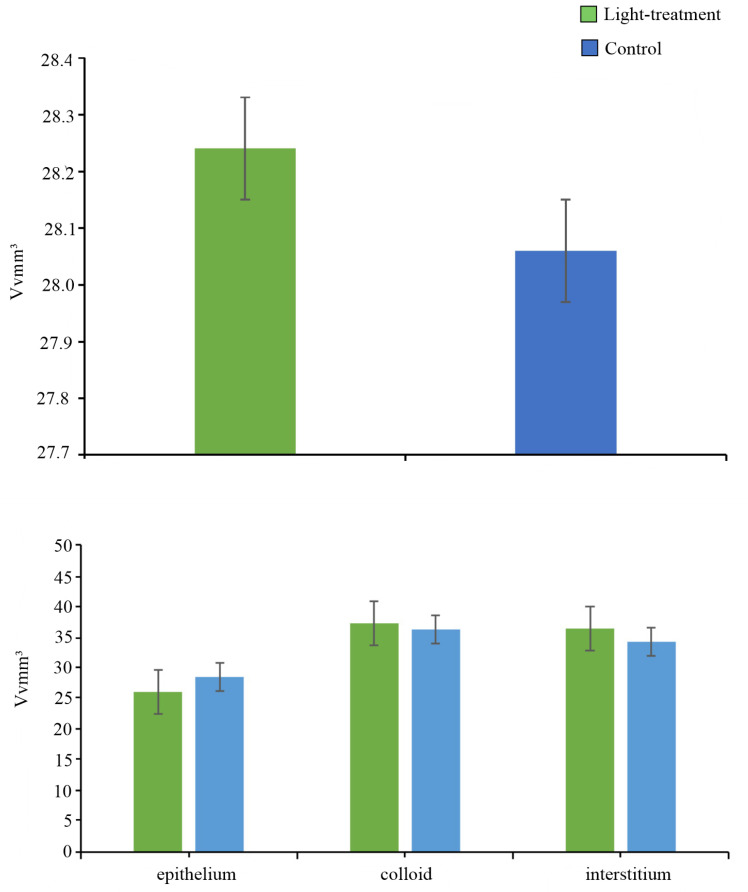
The absolute volume of the thyroid gland (Vv, mm^3^) and volumes of the follicular epithelium, colloid and interstitium (Vv, mm^3^) in the thyroids of light-treatment and control groups (*n* = 6 per group). The values are mean ± SE.

Stereological analysis and results of the Mann–Whitney U test demonstrated that there were no statistically significant differences in the absolute volume density of the whole thyroid gland (*p* = 0.873) and the volumes of the follicular epithelium (*p* = 0.689), colloid (*p* = 0.749) and interstitium (*p* = 0.936) of the thyroid gland between the control and the light-treatment group (Figure 2).

### 3.2. Immunohistochemical Analysis of Tg Immunostaining

**Figure 3:**
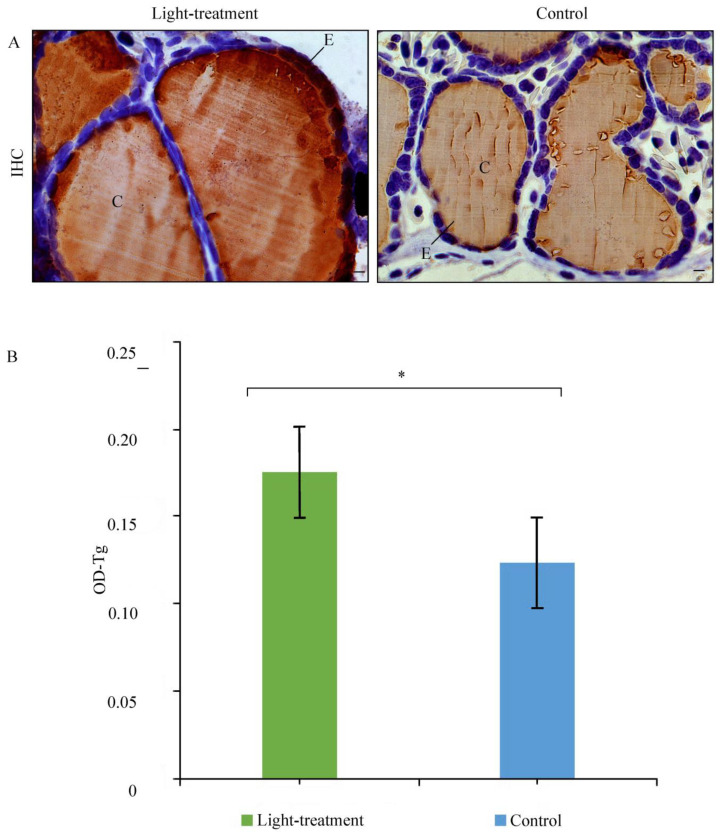
(**A**) Representative micrographs of immunohistochemical staining for thyroglobulin (Tg) in the thyroid gland of *Triturus* newts from the light-treatment and control groups. Abbreviation: C, colloid; E, epithelium; 40× magnification (bar = 50 µm). (**B**) Optical density (OD) of thyroglobulin (Tg) immunopositivity in the thyroid gland of crested newts from the light-treatment and control groups. Values are presented as mean ± SE; asterisks indicate significant differences between light-treatment and control groups (*p* < 0.05).

In the control animals, Tg immunostaining was strong and uniformly distributed within the cytoplasm of thyroid follicular epithelial cells. Weaker Tg immunopositivity was also detected in the luminal colloid (Figure 3A). In animals exposed to light, the pattern of Tg immunostaining remained unchanged (Figure 3A). However, the OD of Tg immunopositivity was higher in light-treatment animals compared to the control group, suggesting that a higher concentration of thyroglobulin in the thyroid gland indicates low thyroid activity (Figure 3A). There was a statistically significant difference in OD between the light-treatment and control groups (*p* = 0.030) (Figure 3B).

## 4. Discussion

Artificial light at night is an emerging environmental stressor that can disrupt amphibian physiology [15,16,17]. By altering photoperiod cues, ALAN may affect thyroid function, growth, and behavior. Despite their sensitivity, amphibians remain largely understudied, though evidence suggests nocturnal illumination elevates stress and alters activity, with potential endocrine and ecological consequences [41,42]. In this study, we investigated whether chronic nighttime exposure to cool-white LED light affects thyroid morphology and thyroglobulin dynamics in metamorphosed juvenile crested newts (*Triturus ivanbureschi*). While absolute thyroid volume and the volumes of the follicular epithelium, colloid, and interstitium did not differ between groups, subtle morphological changes in the thyroid gland were observed. Light-treatment animals were characterized by the presence of macrofollicles and the absence of resorption vacuoles, indicating a less active state of the thyroid gland. Moreover, the thyroid gland of light-treatment animals was characterized by a flattened epithelium and higher OD of Tg, indicating the accumulation of Tg in the thyrocytes with a smaller release into circulation. The observed higher concentration of Tg in the thyroid gland indicates a hypofunctional state in the light-treatment group compared to thyroid glands of the control group. Although the observed differences in the thyroid glands of light-treated animals are relatively subtle when compared with those treated, for example, with thiourea, they indicate possible consequences on their physiological functions. Previous research demonstrated that thiourea, which represents a well-known endocrine disruptor, induces drastic changes in the thyroid gland morphology of *Triturus* newts [27] most likely by inhibiting thyroid peroxidase, blocking THs synthesis, decreasing T_3_/T_4_, and increasing TSH [25,43,44].

Studies in various animal species demonstrate that prolonged or intense light exposure is associated with morphological and functional changes in the thyroid gland, particularly in fish and rodents, highlighting the potential for ALAN to act as an environmental endocrine disruptor [45,46,47,48,49]. The exact mechanisms by which ALAN affects thyroid function remain unclear, as thyroid regulation involves multiple interconnected levels. One plausible pathway is via melatonin suppression. Melatonin secretion normally peaks after midnight, regulating circadian rhythms, neuroendocrine functions, and body temperature cycles [50,51]. Melatonin acts as an inhibitory modulator of the hypothalamic–pituitary–thyroid (HPT) axis and influences thyroid hormone metabolism, physiologically suppressing TH levels during the night [52]. Pineal melatonin secretion is regulated by photoperiod through the suprachiasmatic nucleus, which receives light–dark information from the retina; even brief nocturnal light exposure can disrupt this pathway [52]. Consequently, ALAN may indirectly alter thyroid function by suppressing melatonin production and desynchronizing circadian regulation of the HPT axis, leading to altered TSH secretion, thyroid hormone imbalance, and reduced glandular activity. The increased Tg concentration observed in light-exposed animals in our study supports this mechanism. However, in amphibians, the changes in circadian regulators by ALAN are still poorly understood. For instance, ref. [53] reported only slight effects of low-level ALAN on the melatonin signaling pathway in two anuran species, whereas prolonged exposure to extended photoperiods (18 h light/6 h dark) significantly suppressed nocturnal melatonin production [54].

Another potential mechanism by which ALAN may influence thyroid function is through corticosterone (CORT) and the hypothalamic–pituitary–interrenal (HPI) axis. In post-metamorphic amphibians, CORT often interacts with TH pathways via shared nuclear receptors, affecting metabolism and tissue development [55]. Exposure to different light types and intensities has been shown to significantly alter CORT levels across various amphibian species and life stages [16,56,57]. Elevated CORT can inhibit TSH action on the thyroid gland and reduce T4 to T3 conversion in tissues, resulting in a hypofunctional state. Because thyroid activity is closely linked to metabolic rate, ALAN-induced endocrine disruption can have downstream effects; for example, exposure to 5–20 lux of nighttime light has been shown to reduce standard metabolic rates in common toads [41]. Such disturbances may cascade into reduced growth, altered physiology and behavior, impaired immunity, and ultimately lower fitness and survival in crested newts [58].

Overall, our findings suggest a potential link between ALAN exposure and thyroid hypofunction in post-metamorphic juvenile newts, potentially mediated through endocrine disruption involving melatonin and corticosterone, key regulators of circadian physiology. However, confirmation of these mechanisms requires further investigation at hormonal and molecular levels to fully elucidate the pathways by which ALAN affects thyroid function in amphibians. In this context, a limitation of the present study is the absence of direct measurements of circulating thyroid hormones. In juvenile crested newts, small body size and limited blood volume preclude reliable quantification of circulating thyroid hormones without the use of larger numbers of individuals (e.g., pooled samples) and/or species-specific validated assays. Consequently, the present conclusions are based on thyroid histology and thyroglobulin-related structural indicators, highlighting the need for future studies that integrate hormone measurements with developmental and metamorphic endpoints.

## 5. Conclusions

ALAN is spreading rapidly worldwide, yet its endocrine effects on wildlife, particularly amphibians, remain poorly understood. Our study adds to this growing evidence by demonstrating subtle but consistent changes in the thyroid glands of light-exposed newts. These animals exhibited the presence of macrofollicles, a dense colloid, a flattened epithelium, and higher thyroglobulin levels, all indicative of reduced thyroid activity. Although the observed changes are modest compared with those caused by known endocrine disruptors, they suggest that ALAN can influence amphibian thyroid physiology via different mechanisms. A likely mechanism is that ALAN disrupts circadian regulators, including melatonin and corticosterone, leading to dysregulation of the HPT axis and contributing to the hypofunctional thyroid state observed in light-exposed animals. Overall, our findings highlight ALAN as a potential endocrine disruptor in amphibians and emphasize the need for further studies to clarify its mechanisms and assess the ecological consequences of long-term exposure. Furthermore, incorporating additional larval stages throughout larval development and assessing responses under different lighting conditions may further elucidate the potential cumulative negative impacts of ALAN.

## Data Availability

The raw data supporting the conclusions of this article will be made available by the authors on request.
